# Comprehensive Analysis of the 5xFAD Mouse Model of Alzheimer’s Disease Using dMRI, Immunohistochemistry, and Neuronal and Glial Functional Metabolic Mapping

**DOI:** 10.3390/biom14101294

**Published:** 2024-10-13

**Authors:** Emil W. Westi, Saba Molhemi, Caroline Termøhlen Hansen, Christian Stald Skoven, Rasmus West Knopper, Dashne Amein Ahmad, Maja B. Rindshøj, Aishat O. Ameen, Brian Hansen, Kristi A. Kohlmeier, Blanca I. Aldana

**Affiliations:** 1 Department of Drug Design and Pharmacology, Faculty of Health and Medical Sciences, University of Copenhagen, 2100 Copenhagen, Denmark; emil.westi@sund.ku.dk (E.W.W.); caroline.termohlen@gmail.com (C.T.H.); dash@cfin.au.dk (D.A.A.); maja.rindshoej@gmail.com (M.B.R.); aisha.ameen@sund.ku.dk (A.O.A.); kak1@sund.ku.dk (K.A.K.); 2 Center of Functionally Integrative Neuroscience, Department of Clinical Medicine, Aarhus University, 8000 Aarhus, Denmark; saba.molhemi@cfin.au.dk (S.M.); cskoven@cfin.au.dk (C.S.S.); west@cfin.au.dk (R.W.K.); brian@cfin.au.dk (B.H.); 3 Sino-Danish Center for Education and Research, University of Chinese Academy of Sciences, Beijing 100040, China

**Keywords:** Alzheimer’s disease, 5xFAD mouse, amyloid-beta, gliosis, white mater degeneration, astrocytes, microglia, energy metabolism, diffusion MRI

## Abstract

Alzheimer’s disease (AD) is characterized by complex interactions between neuropathological markers, metabolic dysregulation, and structural brain changes. In this study, we utilized a multimodal approach, combining immunohistochemistry, functional metabolic mapping, and microstructure sensitive diffusion MRI (dMRI) to progressively investigate these interactions in the 5xFAD mouse model of AD. Our analysis revealed age-dependent and region-specific accumulation of key AD markers, including amyloid-beta (Aβ), GFAP, and IBA1, with significant differences observed between the hippocampal formation and upper and lower regions of the cortex by 6 months of age. Functional metabolic mapping validated localized disruptions in energy metabolism, with glucose hypometabolism in the hippocampus and impaired astrocytic metabolism in the cortex. Notably, increased cortical glutaminolysis suggested a shift in microglial metabolism, reflecting an adaptive response to neuroinflammatory processes. While dMRI showed no significant microstructural differences between 5xFAD and wild-type controls, the study highlights the importance of metabolic alterations as critical events in AD pathology. These findings emphasize the need for targeted therapeutic strategies addressing specific metabolic disturbances and underscore the potential of integrating advanced imaging with metabolic and molecular analyses to advance our understanding of AD progression.

## 1. Introduction

Alzheimer’s disease (AD) is a progressive neurodegenerative disorder characterized by cognitive decline and memory loss [1]. Pathologically, AD is defined by the accumulation of amyloid-beta (Aβ) plaques and neurofibrillary tangles (NFTs) in the brain [2,3]. While the exact mechanisms underlying AD pathogenesis remain elusive, mounting evidence suggests that neuroinflammation and cerebrovascular dysfunction play critical roles in disease progression [4,5].

To elucidate the temporal evolution of neuropathological and neuroimaging alterations associated with AD, we employed a comprehensive multimodal approach in the 5xFAD mouse model, a well-established transgenic model that recapitulates key pathological features of human AD. The 5xFAD mouse model is a valuable tool for studying AD due to its rapid and aggressive development of AD-like pathology [1]. By 2 months, these mice exhibit Aβ plaques with intraneuronal Aβ_42_ accumulation, leading to oxidative stress, mitochondrial dysfunction, and synaptic deficits [1]. These early molecular alterations are accompanied by cellular changes, including microgliosis and astrocytosis, which contribute to the neuroinflammatory environment observed in the 5xFAD brain [1]. This stage is akin to the preclinical stage of AD in humans, where amyloid pathology is present without noticeable cognitive symptoms [6]. At 6 months, the pathological landscape in the 5xFAD mouse model becomes more pronounced. There is significant neuronal loss in regions with high amyloid burden, such as the hippocampus and cortex [1,7], as well as synaptic dysfunction and behavioral deficits in tasks like the Morris water maze. These alterations may correspond to the mild cognitive impairment (MCI) stage in humans, where cognitive symptoms become noticeable but do not yet interfere significantly with the patient´s daily life [6]. Metabolic changes, such as altered glucose metabolism and increased oxidative stress, may further exacerbate neuronal damage at this stage. Overall, the progression from 2 to 6 months in 5xFAD mice effectively models the transition from preclinical AD to MCI in humans, enabling studies into early pathological changes and potential therapeutic targets. The present study thus aimed to investigate the spatiotemporal dynamics of Aβ deposition, glial activation, functional brain metabolism, and microstructural integrity in 5xFAD mice at 2 and 6 months of age.

Immunohistochemistry for Aβ, glial fibrillary acidic protein (GFAP) as a marker for astrocytes, and ionized calcium binding adaptor molecule 1 (IBA1) as a marker for microglia was performed to assess amyloid pathology and neuroinflammation, while oligodendrocyte-derived myelin basic protein (MBP) was detected to assess demyelination. To evaluate functional brain metabolism, we utilized dynamic metabolic mapping with heavy stable isotopes and mass spectrometry. Finally, diffusion MRI (dMRI) was employed to assess microstructural changes within the brain.

By combining these complementary techniques, we sought to gain insights into the early pathological processes underlying AD and to identify potential biomarkers for disease progression. Understanding the temporal evolution of these alterations is crucial for developing effective therapeutic interventions and early diagnostic strategies for AD.

## 2. Materials and Methods

### 2.1. Materials

Anesthetics isoflurane, ketamine/xylazine, and saline solution with heparin were from Nomeco, Copenhagen, Denmark). Paraformaldehyde (PFA) was from Th. Geyer, Ballerup, Denmark. Phosphate-buffered saline (PBS), citrate solution, Triton X-100, and mounting media were from Merck, Søborg, Denmark. The stable ^13^C-enriched compounds [U-^13^C]glucose (CLM-1396-5, 99%) and [U-^13^C]glutamine (CLM-1822-H-PK, 99%) were all from Cambridge Isotope Laboratories (Tewksbury, MA, USA), and [1,2-^13^C]acetate (CLM-440-1, sodium salt, 99%) was from ISOTEC (St. Louis, MO, USA). Chemicals otherwise used were of the purest grade available from regular commercial sources.

### 2.2. Animals

All animal experiments and procedures were approved by the Animal Welfare Committee (permit number: 2020-15-0201-00441) appointed by the Danish Ministry of Justice and in accordance with the European Communities Council Directive of 22 September 2010 (2010/63/EU) on the Protection of Animals Used for Experimental and Other Scientific Purposes. Prior to the start of the experiment, mice were randomly allocated to experimental groups (5xFAD vs. control) at the corresponding ages.

Transgenic male 5xFAD mice (TG(APPSwFlLon, PSEN1*M146L*L286V)6799Vas, Jax strain: 006554) and wild-type (WT) females (Jax strain: 100012) on a B6/SJLF1J background were obtained from Jackson Laboratories (Bar Harbor, ME, USA). A breeding colony was maintained at the Department of Drug Design and Pharmacology, University of Copenhagen. The 5xFAD mice express five familial AD mutations in the APP and PSEN1 genes under the Thy1 promoter, leading to cerebral amyloid deposition [8] Mice were housed in individually ventilated cages in a pathogen-free, temperature- (22 ± 2 °C) and humidity- (36–58%) controlled facility with a 12-h light/dark cycle and free access to water and chow. Genotyping was performed using standard PCR (Jax protocol: 23370) on ear and tail clippings for the APP gene.

For immunohistochemistry, 2- and 6-month-old heterozygote 5xFAD mice were compared with age-matched WT controls. Each group consisted of three mice (n = 3 per genotype per condition), mixing females and males based on availability. For functional metabolic mapping, 6-month-old heterozygote 5xFAD mice (corresponding to advanced disease stage) and WT littermates were used as controls (n = 4 mice per genotype). Previous metabolic characterizations in 2-month-old 5xFAD mice compared to WT littermates have been published [9]. For dMRI, 6–7 mice per genotype were used, and only female mice were included in these experimental sets due to higher severity in amyloid pathology development [8].

### 2.3. Immunohistochemistry

**Figure 1:**
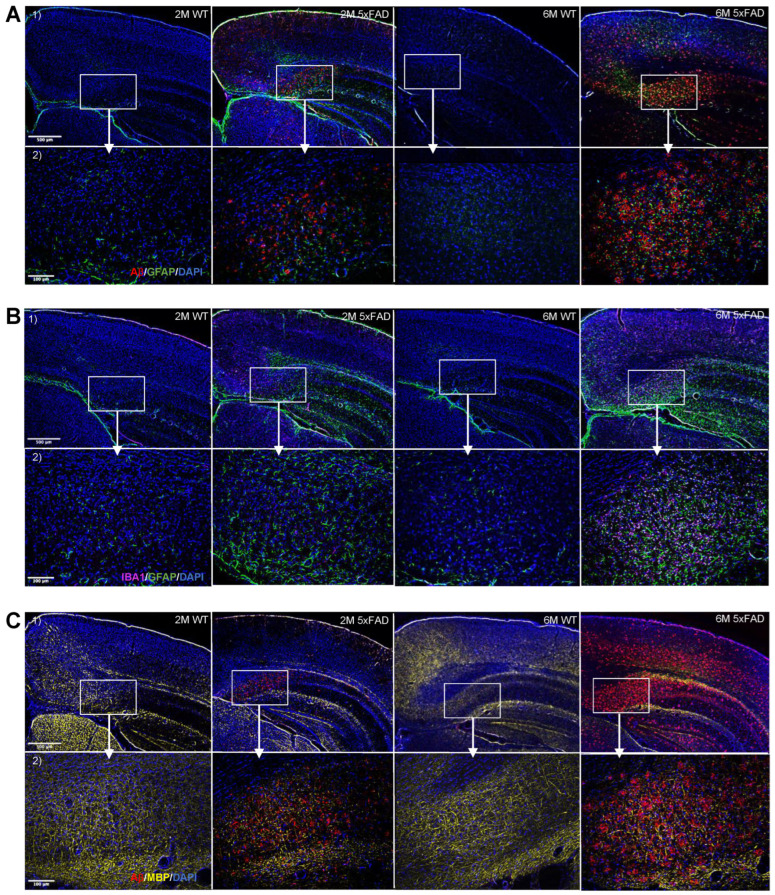
Progressive increase in Aβ accumulation, myelin degeneration, and gliosis in the 5xFAD brains. Representative fluorescence staining images from triple-stainings in brain slices of 5xFAD and WT with (**A**) Aβ/GFAP/DAPI, (**B**) IBA1/GFAP/DAPI, and (**C**) Aβ/MBP/DAPI at 2 (2M) and 6 (6M) month timepoints. (1) 5× magnification (2.02 µm/pixel) microscopy images of brain slices showing subiculum, upper cortex, lower cortex, and white matter. (2) 20× magnification (0.5128 µm/pixel) microscopy images of brain slices showing subiculum. Scale bars indicate 500 µm and 100 µm for upper and lower rows, respectively.

**Figure 2:**
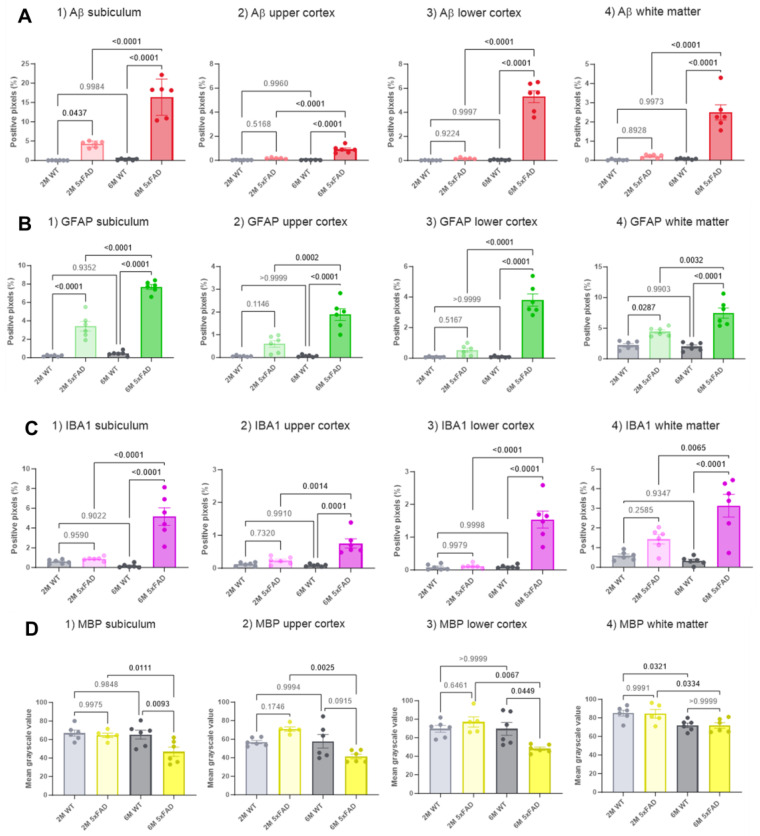
Quantification of pathological markers in the brains of 5xFAD and WT mice at two ages. Fluorescence microscopy images (represented in Figure 1) were quantified, and the corresponding signal derived from the markers (**A**) Aβ, (**B**) GFAP, (**C**) IBA1 and (**D**) MBP is presented. Four different brain regions are shown: (1) subiculum, (2) upper cortex, (3) lower cortex, and (4) white matter. Each bar graph shows populations of the level of markers in 2M 5xFAD, 2M WT, 6M 5xFAD, and 6M WT mice. The bar graphs are presented as mean percentages of either positive pixels (**A**–**C**), or mean gray-scale value (**D**) ± SD. The number of brain slices is 6 (from 3 independent mice per genotype per age). The statistical comparisons (determined with ANOVA and Tukey’s test) are presented with *p* values above the bars, with the significant values in bold.

The mice were anesthetized using isoflurane and ketamine/xylazine and transcardially perfused with heparinized saline followed by 4% PFA in PBS. After decapitation, brains were post-fixed in 4% PFA for 48 h, then cryoprotected in 30% sucrose until cryostat sectioning. Coronal brain slices (40 µm) were cut using a cryostat (CM 3050 S, Leica Microsystems, Wetzlar, Germany) and stored in PBS at 4 °C. Immunofluorescence involved triple-staining for Aβ, GFAP, IBA1, and MBP, with DAPI as a counterstain. Brain slices, selected every 10th section from bregma −4.48 mm to 0.50 mm, were processed over three days. Primary and secondary antibodies were applied sequentially, with all steps conducted under reduced light exposure. Stained slices were mounted on glass slides, dried, and stored at 4 °C. Fluorescence microscopy was performed using an Axioskop 2 microscope (Oberkochen, Germany) with AxioCam MR3 b/w camera. Images were captured and processed in Axiovision 4.8 software. Exposure times were optimized per image to prevent overexposure, and images were taken at 20× magnification across multiple regions of interest (ROI) in the subiculum, cortex, and white matter (Figure 1). Histological quantification was conducted using a custom MATLAB (v. R2022a, MathWorks, Natick, MA, USA) script for image segmentation and processing. Positive pixels for Aβ, GFAP, and IBA1 were quantified as a percentage, while MBP was measured by mean signal intensity across the ROI (Figure 2). DAPI cell counts involved background subtraction, contrast enhancement, and binarization. The MATLAB script is available upon request. For figures, pseudo color was applied, with color applied equally across the entire image. The list of antibodies employed can be found in Supplementary Table S1.

### 2.4. Functional Metabolic Mapping

**Figure 3:**
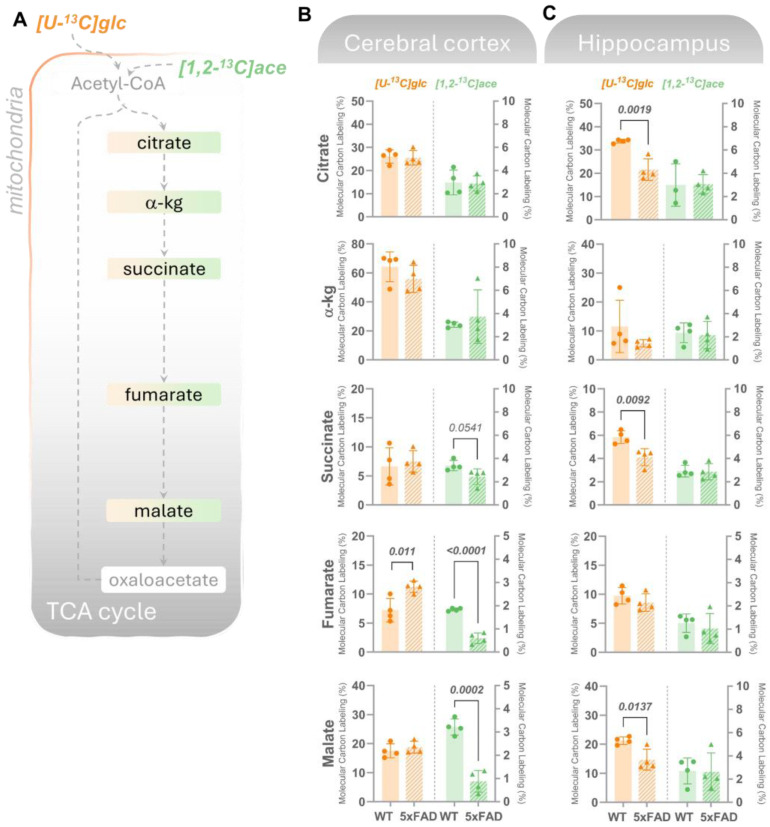
Cortical acetate metabolism and hippocampal glucose metabolism is selectively affected in 6M 5xFAD brains. (**A**) Mitochondrial oxidative metabolism of [U-^13^C]glucose (glc, left axes orange bars) or [1,2-^13^C]acetate (ace, right axes, green bars) gives rise to ^13^C-enrichment (detected by GC–MS) in TCA cycle intermediates in acutely isolated slices from (**B**) cerebral cortex or (**C**) hippocampus incubated with the labeled substrates for 60 min. Molecular carbon labeling (MCL), the weighted average of the carbon labeling in given metabolic intermediates, is presented. [U-^13^C]glucose reflects overall energy metabolism where neurons are the main energy consumers, while [1,2-^13^C]acetate is predominantly metabolized in astrocytes. In the cerebral cortex, overall maintained ^13^C-incorporation in TCA cycle metabolites from [U-^13^C]glucose was observed, while lower MCL in intermediates from [1,2-^13^C]acetate was found in 5xFAD mice compared to wild-type (WT) controls. The opposite was observed for the hippocampal slices, where a lower MCL in intermediates resulting from [U-^13^C]glucose metabolism but maintained [1,2-^13^C]acetate was detected in the 5xFAD mice vs. WT. Values represent mean (±) SD (n  =  4 animals). WT animals are represented as circles, while 5xFAD animals are represented as triangles. The statistical significance (determined with Student’s unpaired *t*-test) is presented with *p* values above the bars, with significant values in bold.

**Figure 4:**
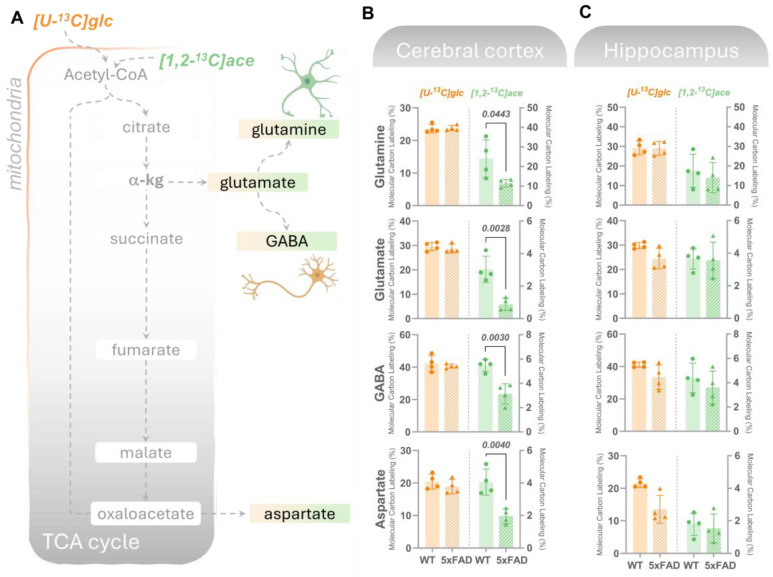
Amino acids derived from acetate metabolism are lower in the cortex but unchanged in the hippocampus in 6M 5xFAD brains. (**A**) Mitochondrial oxidative metabolism of [U-^13^C]glucose (glc, left axes, orange bars) or [1,2-^13^C]acetate (ace, right axes, green bars) gives rise to ^13^C-enrichment (detected by GC–MS) in TCA cycle intermediates and derived amino acids in acutely isolated slices from (**B**) cerebral cortex or (**C**) hippocampus incubated with the labeled substrates for 60 min. Molecular carbon labeling (MCL), the weighted average of the carbon labeling in given metabolic intermediates, is presented. [U-^13^C]glucose reflects overall energy metabolism where neurons are the main energy consumers, while [1,2-^13^C]acetate is predominantly metabolized in astrocytes. In the cerebral cortex, overall maintained ^13^C-incorporation in TCA cycle-derived amino acids from [U-^13^C]glucose was observed, while lower MCL in amino acids from [1,2-^13^C]acetate metabolism was found in 5xFAD mice compared to wild-type (WT) controls. In hippocampal slices, a maintained MCL in amino acids resulting from [U-^13^C]glucose and [1,2-^13^C]acetate metabolism was detected in the 5xFAD mice vs. WT. Values represent mean (±) SD (n  =  4 animals). WT animals are represented as circles, while 5xFAD animals are represented as triangles. The statistical significance (determined with Student’s unpaired *t*-test) is presented with *p* values above the bars, with significant values in bold.

**Figure 5:**
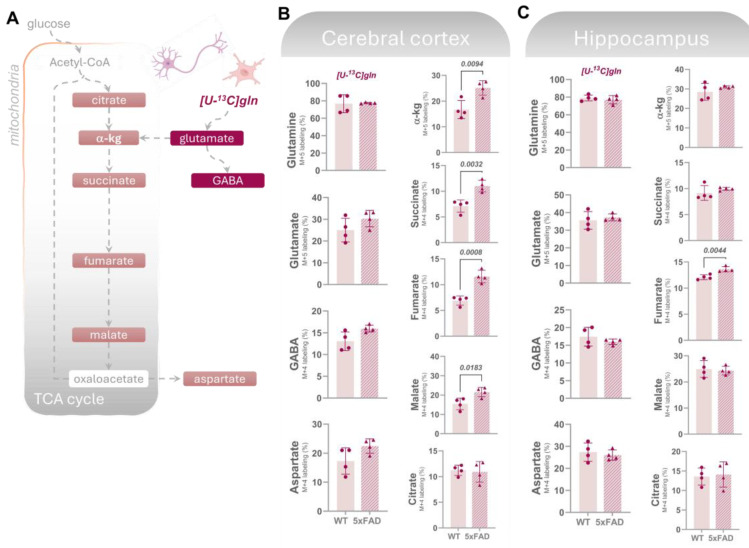
Glutamine uptake is unchanged while its metabolism is higher in the 5xFAD cortex. (**A**) Uptake and metabolism of [U-^13^C]glutamine (gln) gives rise to ^13^C-enrichment (detected by GC–MS) in glutamate, GABA, and TCA cycle intermediates in acutely isolated slices from (**B**) cerebral cortex or (**C**) hippocampus incubated with the labeled substrates for 60 min. ^13^C-enrichment from direct glutamine metabolism is presented as M + X labeling % (X = number of ^13^C-carbons in a given molecule). In the cerebral cortex, overall maintained ^13^C-incorporation in amino acids from [U-^13^C]gln was observed, while higher labeling was detected in most TCA cycle intermediates in 5xFAD mice compared to wild-type (WT) controls, suggesting increased glutaminolysis. In hippocampal slices, a maintained labeling in amino acids and TCA cycle intermediates resulting from [U-^13^C]gln metabolism, with the exception of fumarate, was detected in the 5xFAD mice vs. WT. Values represent mean (±) SD (n  =  4 animals). WT animals are represented as circles, while 5xFAD animals are represented as triangles. The statistical significance (determined with Student’s unpaired *t*-test) is presented with *p* values above the bars, with significant values in bold.

The brain slice incubations and metabolic mapping using heavy stable isotopes and Gas Chromatography–Mass Spectrometry (GC–MS) analyses were performed as previously reported [10]. Briefly, female mice at 6 months of age were euthanized by cervical dislocation, and the brains were rapidly excised and placed in ice-cold artificial cerebrospinal fluid (ACSF; pH 7.4) containing in millimolar: NaCl, 128; NaHCO_3_, 25; D-glucose, 10; KCl, 3; CaCl_2_, 2; MgSO_4_, 1.2; KH_2_PO_4_, 0.4. The cerebral cortex and hippocampus were isolated in ice-cold ACSF. Brain slices (350 µm) were prepared using a McIlwain tissue chopper and pre-incubated in oxygenated (5% CO_2_/95% O_2_) ACSF at 37 °C for 60 min. Slices were then incubated for an additional 60 min in ACSF containing ^13^C-enriched substrates: 2.5 mM [U-^13^C]glucose, 5 mM [1,2-^13^C]acetate, or 1.0 mM [U-^13^C]glutamine, including 5 mM unlabeled D-glucose. The process was terminated by transferring slices to ice-cold 70% ethanol. Tissues were sonicated, centrifuged, and the supernatants lyophilized for further analysis. Lyophilized brain slice extracts were analyzed by GC–MS to determine ^13^C enrichment in tricarboxylic acid (TCA) cycle intermediates and related amino acids as previously described in detail [11]. Extracts were reconstituted, acidified, extracted with ethanol, and derivatized. Samples were analyzed using GC (Agilent 7820A, Santa Clara, CA, USA) coupled to MS (Agilent 5977E). Isotopic enrichment data were corrected for natural ^13^C abundance, with results presented as molecular carbon labeling (MCL, Figure 3 and Figure 4). MCL quantifies ^13^C incorporation into metabolites within cellular pathways [12]. Data in Figure 5 are presented as a percentage of labeling of the isotopologue M  +  X, where M equals the molecular weight of the unlabeled molecule, and X is the number of ^13^C-enriched carbon atoms [13].

### 2.5. Diffusion Magnetic Resonance Imaging (dMRI)

#### 2.5.1. Sample Preparation

The brains were scanned in-skull to avoid brain deformation as previously described [14,15]. Before imaging, the samples were briefly rinsed in potassium phosphate buffered saline (KPBS) and then washed in fresh KPBS for at least 24 h on a benchtop shaker at room temperature. This was done to increase the signal by removal of excess fixative [16]. Similar to previous studies [17,18,19,20,21], the samples were subsequently mounted in a 15 mL MRI-compatible Falcon tube filled with a perfluorocarbon-based liquid (Fluorinert, 3M, PN: FC-770 Merck, Søborg, Denmark). Consistent positioning of the samples and reduced presence of bubbles close to the sample throughout the experiments were ensured by an in-house 3D-printed (Prusa MK3+; Prusa Research, Prague, Czech Republic) sample holder and bubble trap (PolyLactic Acid, 3DE Premium, 3D Eksperten, Norresundby, Denmark), fitted for the tube.

#### 2.5.2. MRI Data Collection

MRI was performed using a 9.4 T preclinical system (BioSpec 94/20, Bruker Biospin, Ettlingen, Germany) equipped with a bore-mounted 25 mm quadrature transmit-receive coil. To reduce sample vibrations, the tube containing the sample was fitted into the coil using a custom-shaped polyethylene foam cylinder. Optimal sample placement was achieved with initial localizer scans having a field of views (FOVs) of 51.2 mm × 52.1 mm, and 19.2 mm × 19.2 mm, respectively. Scan time: ∼0.5 min. To improve echo-planar imaging (EPI), we obtained a high-resolution B0-field map. The scan parameters were echo time (TE): 1.84 ms, echo spacing: 3.57 ms, repetition time (TR): 20 ms. Matrix size: 128 × 128 × 128, FOV: 51.2 mm × 51.2 mm × 51.2 mm—resulting in a spatial resolution of 0.25 mm × 0.25 mm × 0.25 mm. Scan time: ∼6 min. Both structural data and DKI data were acquired for each sample. Structural data were acquired using a T2-weighted turbo rapid acquisition with relaxation enhancement (RARE) sequence with 50 μm ×50 μm in-plane resolution, and 60 slices with a thickness of 200 μm. FOV: 17 mm × 9 mm × 16 mm. The remaining scan parameters used were effective TE: 10.6 ms, TR: 4856 ms, 30 averages and a RARE factor: 2. Scan time: ∼3 h 39 min.

The DKI data were acquired using a diffusion-weighted spin-echo EPI sequence with 8 segments, a 150 μm × 150 μm in-plane resolution, and 60 slices of 200 μm thickness (same slice positions as the RARE data described above). Five unweighted A0 images were acquired for normalization of the 30 isotropically distributed diffusion encoding directions at each of the four non-zero b-values, b = [0.4, 0.8, 1.5, 2.0] ms/μm^2^. Additional scan parameters were duration of diffusion gradients (δ) = 8 ms, diffusion gradient separation (∆) = 18 ms, 20 averages, TE = 32.57 ms, TR = 4000 ms, bandwidth ∼278 kHz, with a scan time of ∼22 h 14 min. Total scan time: ∼26 h.

#### 2.5.3. DKI Analysis

For analysis, DKI data were preprocessed as described previously [22]. Briefly, data were denoised [23], Rician noise floor adjusted [24], and corrected for Gibbs ringing [25]. The data were then fitted to the DKI signal equation using non-linear optimization (MATLAB) as in [26]. From the DKI fit, the mean diffusivity (MD), fractional anisotropy (FA), and mean kurtosis (MK) were calculated for each sample [27]. For analysis, regions of interest (ROIs) were manually drawn as described below.

#### 2.5.4. Image Segmentation

All ROIs were manually outlined in 2D coronal cross-sections from the T2-weighted images, using ITK-SNAP [28] aided by standard mouse brain atlases [29,30]. The ROIs were: neocortex, upper and lower cortical layer, subiculum, and a general WM-region. To avoid partial volume effects, this WM-region consisted of corpus callosum, cingulum, external capsule and the dorsal hippocampal commissure (Figure S1 in Supplementary Materials). Next, the ROIs were imported into MATLAB (The Mathworks) where the values of MD, MK, and FA within each ROI mask were extracted for further analysis.

### 2.6. Statistical Analyses

Statistical analyses for immunohistochemistry were conducted using GraphPad Prism 9.3.1 (GraphPad Software, San Diego, CA, USA). A robust regression and outlier removal (ROUT) test (Q = 1%) identified and removed one outlier. For comparisons across four groups with two independent variables, a two-way analysis of variance (ANOVA) was used without prior normality testing, as this test is robust to normality violations (AMSTAT Consulting, 2005, Palo Alto, CA, USA). Significant results were followed by Tukey’s post hoc test. For comparisons between two groups, a paired Student’s *t*-test was applied, with normality confirmed by the Shapiro–Wilk test (*p* ≤ 0.05). Statistical comparisons are indicated as *p* values on top of the compared graphs, where *p* ≤ 0.05 denotes significance. Immunohistochemistry data are presented as mean ± SD (standard deviation), with individual datapoints from independent slices (5–6) from 3 mice per genotype per condition. Aβ, GFAP, and IBA1 are expressed as the percentage of positive pixels, MBP as mean grayscale value, and DAPI as cell count/mm². Microscopy images were processed using ImageJ v. 2.9.0/1.53t.For metabolic mapping, data were analyzed in GraphPad Prism 9.3.1, presented as means ± SD with individual data points representing biological replicates (n = independent animals). Comparisons between two groups (5xFAD vs. controls) were made using Student’s unpaired *t*-test. Normality was assessed using D’Agostino–Pearson and Shapiro–Wilk tests, but due to small sample sizes, parametric tests assuming Gaussian distribution were applied. For these data sets, statistical comparisons are also indicated as *p* values on top of the compared graphs, where *p* ≤ 0.05 denotes significance

The statistical analysis for the dMRI was carried out blinded with respect to the genotype. One sample (2M WT) was excluded from the analysis due to sample fixation failure revealed by MRI. Both hemispheres of each region of interest (ROI) were pooled. The pooled ROI volume was normalized to the whole-brain volume to adjust for ROIs scaling with whole-brain size [31]. Both volumetrics and DKI parameters were analyzed using a permutational three-way mixed ANOVA using 10,000 repetitions [32]. Statistically significant interactions were further analyzed using simple main effects analyses. Genotype and age were between-subject factors, while the ROIs were treated as a within-subject factor. The *p*-value was given as the proportion of permutated F-values larger than the observed F-value divided by the number of permutations. The error bars represent 95% confidence intervals (CIs). The graphs were made in python using the matplotlib [33] library and the statistical analysis was carried out in R v.4.4.1 using the “permuco” [34] and “wPerm” [35] packages.

## 3. Results

### 3.1. Age-Dependent Increases in Aβ, GFAP, and IBA1 Levels in the 5xFAD Model Compared to WT Mice, with Marked Differences across Various Brain Regions

Pathological indicators, including Aβ accumulation and heightened expression of glial markers, were assessed to validate the disease hallmarks in the 5xFAD mouse model compared to WT littermates. To this aim, immunohistochemical staining was performed to compare the levels of Aβ, GFAP, IBA1, MBP, and DAPI in the hippocampal region (subiculum), upper and lower cerebral cortex, and white matter of 2-month- (2M) and 6-month- (6M) old mice (Figure 1A).

#### 3.1.1. The Progression in the Aβ Accumulation Is Region-Specific in the 5xFAD Brain

At 2 months, 5xFAD mice showed significantly higher levels of Aβ in the subiculum compared to WT controls. By 6 months, the 5xFAD mice exhibited significantly elevated Aβ levels in the subiculum, lower cortex, upper cortex, and white matter compared to WT mice. Fluorescence microscopy images (Figure 1A) illustrate this progression. The accumulation of Aβ increased with age in the 5xFAD mice, demonstrating a higher level in 6M mice compared to 2M mice, as expected. A post hoc Tukey’s test for multiple comparisons revealed significant differences in the percentages of positive pixels between 2M 5xFAD and 2M WT mice (*p* = 0.0437, with averages of 4.2 ± 0.3% and 0.03 ± 0.01%, respectively) and between 6M 5xFAD and 6M WT mice (*p* < 0.0001, with averages of 16.3 ± 1.9% and 0.3 ± 0.08%, respectively), as shown in Figure 2(A1). Additionally, a significant difference was observed between 6M 5xFAD and 2M 5xFAD mice (*p* < 0.0001, with averages of 16.4 ± 1.9% and 4.2 ± 0.3%, respectively), but not between 2M WT and 6M WT mice (*p* = 0.9984, with averages of 0.03 ± 0.01% and 0.3 ± 0.08%, respectively).

Aβ levels in the cerebral cortex showed a significant difference in both the upper and lower regions, as confirmed by two-way ANOVA tests (upper cortex: *p* < 0.0001; lower cortex: *p* < 0.0001, Figure 2(A2,3). A Tukey’s post hoc test indicated no significant difference in Aβ levels between 2M 5xFAD and 2M WT mice in both regions (upper cortex: *p* = 0.5168; lower cortex: *p* = 0.9765). However, 6M 5xFAD mice showed significantly higher Aβ levels than 6M WT mice in both the upper (*p* < 0.0001) and lower cortex (*p* < 0.0001) (upper cortex: average positive pixels: 6M 5xFAD: 0.9 ± 0.1%, 6M WT: 0.02 ± 0.004%; lower cortex: average positive pixels: 6M 5xFAD: 5.3 ± 0.5%, 6M WT: 0.06 ± 0.01%). Moreover, the 6M 5xFAD mice exhibited higher Aβ levels compared to 2M 5xFAD mice in both cortex regions (upper cortex: *p* < 0.0001, lower cortex: *p* < 0.0001). No significant difference was observed between 2M WT and 6M WT mice in both regions (upper cortex: *p* = 0.9960, lower cortex: *p* = 0.9997). Although Aβ distribution appeared similar between the groups in both parts of the cortex, a significant difference was noted between the upper and lower cortex of 6M 5xFAD mice. A paired Student’s *t*-test indicated higher Aβ levels in the lower cortex compared to the upper cortex of 6M 5xFAD mice (*p* = 0.0003), while no significant difference was found between these regions in 2M 5xFAD mice (*p* = 0.9468) (Figure 2(A2,3)).

A two-way ANOVA for the data obtained from the white matter also showed significant differences in Aβ levels among the groups (*p* < 0.0001) (Figure 2(A4)). A Tukey’s post hoc test revealed no significant difference between 2M 5xFAD and 2M WT mice (*p* = 0.8597. Average positive pixels: 2M 5xFAD: 0.2 ± 0.04%, 2M WT: 0.02 ± 0.01%), but a significantly higher level of Aβ was seen in 6M 5xFAD compared to 6M WT (*p* < 0.0001. Average positive pixels: 6M 5xFAD: 2.5 ± 0.4%, 6M WT: 0.08 ± 0.01%) and between 6M 5xFAD and 2M 5xFAD mice (*p* < 0.0001). No difference was seen between 2M WT and 6M WT mice (*p* = 0.9965).

#### 3.1.2. Glial Activation and Demyelination Are Prominent in the Hippocampus and Cerebral Cortex in the 6M 5xFAD Mice

Fluorescent microscopy images of GFAP staining (Figure 1A,B) showed that 5xFAD mice exhibited higher levels of GFAP in the subiculum and white matter compared to WT in 2M mice, and in the subiculum, upper cortex, lower cortex, and white matter compared to WT in 6M mice. The level of GFAP was also higher in 6M 5xFAD mice compared to 2M 5xFAD mice. In the subiculum (Figure 2(B1)), post hoc Tukey’s tests confirmed significant differences in GFAP levels between 2M 5xFAD and 2M WT mice (*p* < 0.0001. Average positive pixels: 2M 5xFAD: 3.4 ± 0.5%, 2M WT: 0.2 ± 0.03%), and between 6M 5xFAD and 6M WT mice (*p* < 0.0001. Average positive pixels: 6M 5xFAD: 7.7 ± 1.9%, 6M WT: 0.5 ± 0.1%). Additionally, 6M 5xFAD mice showed higher GFAP levels than 2M 5xFAD mice (*p* < 0.0001), with no significant difference between 2M WT and 6M WT mice (*p* = 0.9352). GFAP levels in the cortex also showed significant differences among the groups (upper cortex: *p* < 0.0001; lower cortex: *p* < 0.0001) (Figure 2(B2,3)). A Tukey’s post hoc test revealed no significant differences between 2M 5xFAD and 2M WT mice in either region (upper cortex: *p* = 0.1146. Average positive pixels: 2M 5xFAD: 0.6 ± 0.1%, 2M WT: 0.06 ± 0.01%; lower cortex: *p* = 0.5167. Average positive pixels: 2M 5xFAD: 0.5 ± 0.1%, 2M WT: 0.07 ± 0.01%). However, significant differences were observed between 6M 5xFAD and 6M WT mice in both the upper and lower cortex (upper cortex: *p* < 0.0001. Average positive pixels: 6M 5xFAD: 1.9 ± 0.3%, 6M WT: 0.07 ± 0.02%; lower cortex: *p* < 0.0001. Average positive pixels: 6M 5xFAD: 3.8 ± 0.4%, 6M WT: 0.08 ± 0.02%). Moreover, 6M 5xFAD mice had higher GFAP levels compared to 2M 5xFAD mice in both regions (upper cortex: *p* = 0.0002, lower cortex: *p* < 0.0001). No significant differences were found between 2M WT and 6M WT mice. A paired Student’s *t*-test showed significantly higher GFAP levels in the lower cortex compared to the upper cortex in 6M 5xFAD mice (*p* = 0.0008), but not in 2M 5xFAD mice (*p* = 0.1712). Two-way ANOVA for GFAP in the white matter showed significant differences among the groups (*p* < 0.0001), with higher levels in 6M 5xFAD compared to 2M 5xFAD (Tukey’s test, *p* = 0.0032) and 6M WT mice (Tukey’s test, *p* < 0.0001, average positive pixels: 2M 5xFAD: 4.4 ± 0.3%, 2M WT: 2.2 ± 6M 5xFAD: 7.5 ± 0.8, 6M WT: 2.0 ± 0.3%) (Figure 2(B4)).

Fluorescence microscopy images of IBA1 staining (Figure 1B) indicated higher IBA1 levels in the subiculum, upper cortex, lower cortex, and white matter of 6M 5xFAD mice compared to WT, but no difference at 2M. The level of IBA1 increased with age in 5xFAD mice. Two-way ANOVA and post hoc Tukey’s tests showed significant differences in IBA1 levels in the subiculum among the groups (*p* < 0.0001). No significant difference was found between 2M 5xFAD and 2M WT mice, but significant differences were observed between 6M 5xFAD and 6M WT mice (*p* < 0.0001. Average positive pixels: 6M 5xFAD: 5.2 ± 0.09%, 6M WT: 0.2 ± 0.08%), and between 6M 5xFAD and 2M 5xFAD mice (*p* < 0.0001. Average positive pixels: 2M 5xFAD: 0.9 ± 0.08%). No difference was found between 2M WT and 6M WT mice (Figure 2(C1)). The levels of IBA1 in the cortex also showed significant differences (upper cortex: *p* < 0.0001; lower cortex: *p* < 0.0001). A Tukey’s post hoc test showed no significant differences between 2M 5xFAD and 2M WT mice in both regions, but significant differences were found between 6M 5xFAD and 6M WT mice (upper cortex: *p* < 0.0001. Average positive pixels: 6M 5xFAD: 0.6 ± 0.1%, 6M WT: 0.09 ± 0.01%; lower cortex: *p* < 0.0001. Average positive pixels: 6M 5xFAD: 1.5 ± 0.3%, 6M WT: 0.1 ± 0.02%) and between 6M 5xFAD and 2M 5xFAD mice in both regions (upper cortex: *p* = 0.0014. Average positive pixels: 2M 5xFAD: 0.2 ± 0.05%; lower cortex: *p* < 0.0001. Average positive pixels: 2M 5xFAD: 0.1 ± 0.03%). No significant differences were observed between 2M WT and 6M WT mice. Paired Student’s *t*-tests revealed significant differences in IBA1 levels between the upper and lower cortex of 2M 5xFAD mice (*p* = 0.0334) and 6M 5xFAD mice (*p* = 0.0308) (Figure 2(C2,3)). Two-way ANOVA for IBA1 in the white matter showed significant differences among the groups (*p* < 0.0001), with higher levels in 6M 5xFAD mice (average positive pixels: 3.1 ± 0.6) compared to 2M 5xFAD (*p* = 0.0065. Average positive pixels: 1.4 ± 0.2%) and 6M WT mice (*p* < 0.0001. Average positive pixels: 0.3 ± 0.08%) (Figure 2(C4)). Together, the observed patterns of higher expression of astrocyte and microglia markers (GFAP and IBAI, respectively) in the 5xFAD brains strongly support the age-dependent progression of gliosis in parallel with plaque deposition.

Fluorescent microscopy images of MBP (Figure 1C) revealed that 6-month-old 5xFAD mice exhibit a lower level of MBP in the subiculum and lower cortex compared to WT mice. In 2-month-old mice, no difference in MBP levels was observed between 5xFAD and WT, as shown in Figure 2D. Two-way ANOVA test showed a significant difference in MBP intensity in the subiculum among the groups (2M 5xFAD, 2M WT, 6M 5xFAD, and 6M WT) with a *p*-value of 0.0027. A post hoc Tukey’s test revealed that 6M 5xFAD mice had significantly lower mean grayscale values of MBP compared to 6M WT mice (*p* = 0.0093, with average values of 46.8 ± 5.0 and 65.3 ± 4.8, respectively) (Figure 2(D1)). No significant difference was found between 2M 5xFAD and 2M WT mice (*p* = 0.9975). Furthermore, 6M 5xFAD mice had significantly lower MBP intensity compared to 2M 5xFAD mice (*p* = 0.0111), while no significant differences were observed between 2M WT and 6M WT mice (*p* = 0.9848). In the cerebral cortex (Figure 2 (D2,3)), two-way ANOVA tests for MBP showed significant differences between groups in both the upper (*p* = 0.0046) and lower cortex (*p* = 0.0075). A Tukey’s test indicated no significant differences in the intensity of MBP between 2M 5xFAD and 2M WT. However, MBP intensity was significantly lower in 6M 5xFAD mice compared to 2M 5xFAD mice in both the upper (*p* = 0.0025) and lower cortex (*p* = 0.0067). (Upper cortex average mean grayscale value: 2M 5xFAD: 71.0 ± 2.2, 6M 5xFAD: 41.4 ± 2.6; lower cortex average mean grayscale value: 2M 5xFAD: 77.0 ± 5.6. 6M 5xFAD: 49.9 ± 2.0). Additionally, a significant reduction in MBP intensity was found in the lower cortex of 6M 5xFAD compared to 6M WT mice (*p* = 0.0449 average mean grayscale value: 6M 5xFAD: 49.9 ± 2.0, 6M WT: 69.6 ± 6.9), whereas no significant difference was observed in the upper cortex (*p* = 0.0915). Analysis of MBP intensity in the white matter via two-way ANOVA revealed significant differences among the groups (*p* = 0.0054). A Tukey’s test showed a significant reduction in MBP intensity in 6M 5xFAD compared to 2M 5xFAD mice (*p* = 0.0334 average mean grayscale value: 2M 5xFAD: 84.4 ± 4.6, 6M 5xFAD: 71.8 ± 3.0) and in 6M WT compared to 2M WT mice (*p* = 0.0321 average mean grayscale value: 2M WT: 85.1 ± 3.1, 6M WT: 72.0 ± 2.4) (Figure 2(D4)). No significant differences were found between 2M 5xFAD and 2M WT (*p* = 0.9991) or between 6M 5xFAD and 6M WT mice (*p* > 0.9999).

DAPI staining showed no visual differences between 5xFAD and WT mice in fluorescence microscopy images (Figure 1 and Figure S2). A two-way ANOVA test for DAPI cell count in the subiculum showed no significant differences among the groups (*p* = 0.3346). In the cortex, two-way ANOVA tests showed significant differences in DAPI cell count in both the upper (*p* < 0.0001) and lower cortex (*p* = 0.0055). A Tukey’s test indicated a significantly higher number of DAPI cells/mm^2^ in 2M 5xFAD mice compared to 2M WT mice (*p* = 0.0046. Average cell count/mm^2^: 2M 5xFAD: 4212 ± 106, 2M WT: 5168 ± 73), in 6M 5xFAD compared to 2M 5xFAD (*p* < 0.0001. Average cell count/mm^2^: 2M 5xFAD: 4212 ± 106, 6M 5xFAD: 5913 ± 98) and in 6M WT compared to 2M WT (*p* < 0.0001. Average cell count/mm^2^: 2M WT: 5168 ± 73, 6M WT: 6612 ± 319). No significant difference in the cell count/mm^2^ of DAPI was seen in the upper cortex between 6M 5xFAD and 6M WT (*p* = 0.0608. Average cell count/mm^2^: 6M 5xFAD: 5913 ± 98, 6M WT: 6612 ± 319). In the lower cortex, a significant increase was observed in the 6M WT compared to 2M WT (*p* = 0.0035. Average cell count/mm^2^: 2M WT: 4446 ± 119, 6M WT: 5343 ± 305). In the white matter, two-way ANOVA showed a significant difference in DAPI cell count (*p* = 0.0061). A Tukey’s test revealed a higher number of DAPI cells/mm^2^ in 6M 5xFAD compared to 2M 5xFAD (*p* = 0.0197), with no significant differences between other groups. Since DAPI staining does not distinguish between live and dead cells, further exploration is needed to determine changes in the ratio of live to dead cells.

In summary, these results demonstrate that the expression levels of Aβ, GFAP, and IBA1 vary significantly with age and brain region in 5xFAD mice, revealing a progressive increase in Aβ accumulation and glial activation (indicated by GFAP and IBA1 expression) as the mice age. This progression is particularly evident in the hippocampus and cortex, suggesting these areas are more susceptible to amyloid pathology and related neuroinflammatory responses.

### 3.2. Localized Cell-Specific Energy Metabolism Shifts in the 5xFAD Mice

#### 3.2.1. Metabolism of Glucose and Acetate Is Selectively Affected in the 5xFAD Brain Slices

Our observations featured progressive glial alterations accompanying Aβ deposition, which led us next to investigate whether changes in pathological markers correlate with specific abnormalities in cerebral energy metabolism. Considering that we had previously identified mild metabolic abnormalities in 2-month-old (2M) 5xFAD mice [9] and that the most pronounced alterations in pathological markers observed here were in 6M 5xFAD mice compared to 2M mice, we performed functional metabolic mapping in 6M mice. We thus incubated acutely isolated cerebral, cortical, and hippocampal slices with ^13^C-enriched energy substrates to functionally map the cellular energy metabolism. In the first experimental set, the slices were incubated with [U-^13^C]glucose, which is metabolized through glycolysis to pyruvate. This results in ^13^C labeling in lactate and alanine (M + 3), which remained unchanged in the cortical slices but was reduced in the hippocampus (Figure S3). This indicates sustained glycolytic activity in the 5xFAD cerebral cortical slices but reduced glycolytic flux in the 5xFAD hippocampal tissue. Further oxidation of [U-^13^C]glucose leads to ^13^C accumulation (presented as the molecular carbon labeling, MCL) in TCA cycle intermediates (Figure 3A) and associated amino acids (Figure 4A). Figure 3B shows that in the cerebral cortex, ^13^C-enrichment in most of the studied TCA cycle intermediates derived from the mitochondrial metabolism of [U-^13^C]glucose, including citrate, a-ketoglutarate, succinate, and malate, was not significantly different between WT and 5xFAD mice. In contrast, in the hippocampal slices (Figure 3C), ^13^C-labeling in citrate, succinate, and malate derived from [U-^13^C]glucose was significantly lower in the 5xFAD compared to WT mice. These observations suggest that while glucose metabolism may be maintained in the 5xFAD cerebral cortex at 6M, a marked hypometabolism of glucose is prominent in the hippocampus.

Next, brain slices were provided with [1,2-^13^C]acetate, a substrate primarily entering astrocytic energy metabolism [36]. [1,2-^13^C]acetate enters the mitochondrial TCA cycle via its conversion to acetyl-CoA and condensation with oxaloacetate to produce citrate (Figure 3A). In cortical slices, a significant lower ^13^C-enrichment in succinate, fumarate, and malate in the 5xFAD mice compared to WT was observed, indicating impaired astrocyte TCA cycle activity. Notably, [1,2-^13^C]acetate metabolism was unchanged in hippocampal cortical slices from 5xFAD mice. These findings demonstrate region-specific shifts in brain energy metabolism in 5xFAD mice.

Since neurotransmission is tightly coupled to cellular metabolism [37,38], we subsequently assessed if the observed changes in glucose and acetate metabolism could influence (neuroactive) amino acids. In both cortical and hippocampal slices (Figure 4B,C), no statistical changes were detected in the MCL in glutamate, glutamine, GABA, and aspartate derived from [U-^13^C]glucose. In line with the patterns observed in [1,2-^13^C]acetate-derived TCA cycle intermediates, a significantly lower ^13^C-incorporation in the amino acids was found in the 5xFAD cortical slices incubated with [1,2-^13^C]acetate (Figure 4B), while no changes were found in the 13C-incorporation in amino acids in the hippocampus of 5xFAD mice compared to WT controls (Figure 4C).

#### 3.2.2. Altered Glutaminolysis Dynamics in 5xFAD Cerebral Cortex

Glutamine, exclusively synthesized in astrocytes, is released and taken up by neurons, serving as a crucial substrate for replenishing the glutamate and GABA pools [38]. The enzyme phosphate-activated glutaminase (PAG) initiates glutamine metabolism by converting it into glutamate. Glutamate carbon atoms can be incorporated in TCA cycle intermediates via α-kg or be converted to GABA in GABAergic neurons (Figure 5A). Additionally, it has recently been shown that activated microglia can meet their metabolic demand via increased glutaminolysis [39]. To investigate potential alterations in the cerebral metabolism of glutamine in the 5xFAD mice, we incubated cortical and hippocampal slices with [U-^13^C]glutamine (M + 5). We observed no change in the labeling in glutamine (M + 5) from incubation with [U-^13^C]glutamine, suggesting an unchanged glutamine uptake capacity in the 5xFAD cortical and hippocampal slices (Figure 5B,C). Notably, we found higher ^13^C-enrichment (M + 5/M + 4) in all derived metabolites (with the exception of citrate), but not in the derived amino acids from the direct metabolism of [U-^13^C]glutamine in the cerebral cortical slices of 5xFAD mice (Figure 5A). However, this increase was not observed in the 5xFAD hippocampal slices, where the labeling in amino acid and TCA cycle intermediates was largely unchanged compared to WT slices. This is not the case for fumarate where a higher labeling is observed in the 5xFAD hippocampus. This increase in glutaminolysis in 5xFAD cortex contrasts with the maintained glucose metabolism that could be reflecting overall neuronal metabolism suggesting that activated microglia in 5xFAD mice may display increased glutaminolysis, thereby increasing the availability of key metabolites for neuronal function.

### 3.3. Diffusion MRI (dMRI)

**Figure 6:**
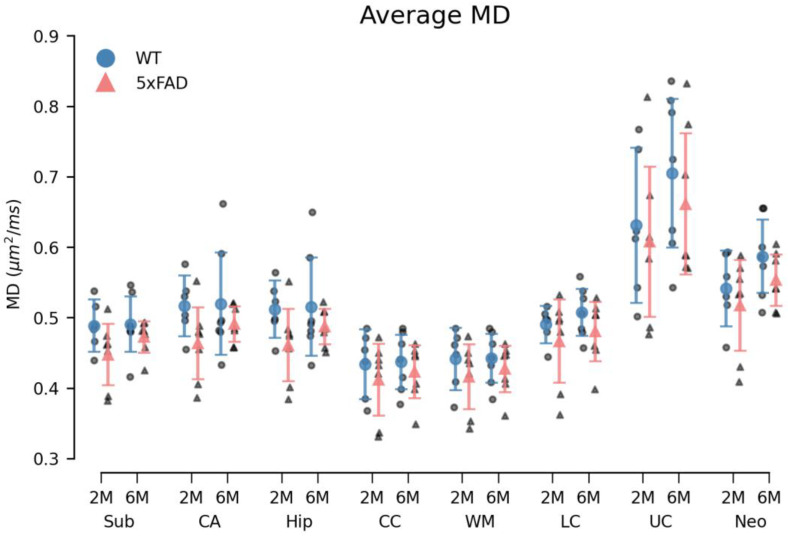
The average mean diffusivity (MD) within each region of interest (ROI) was decreased in 5xFAD brains of both age groups. The MD was found consistently decreased in all ROIs of 5xFAD brains compared to WT brains. However, this difference between genotypes was not statistically significant. Additionally, while a small age-related increase in MD was observed in all ROIs for both genotypes, this difference was mainly evident in 5xFAD brains. Group means with 95% confidence intervals are superimposed on the individual observations. 2M = 2-month-old, 6M = 6-month-old. Sub = subiculum, CA = cornu ammonis of the hippocampal formation, hip = hippocampus, CC = corpus callosum, WM = white matter, LC = lower cortex, UC = upper cortex, and neo = neocortex.

**Figure 7:**
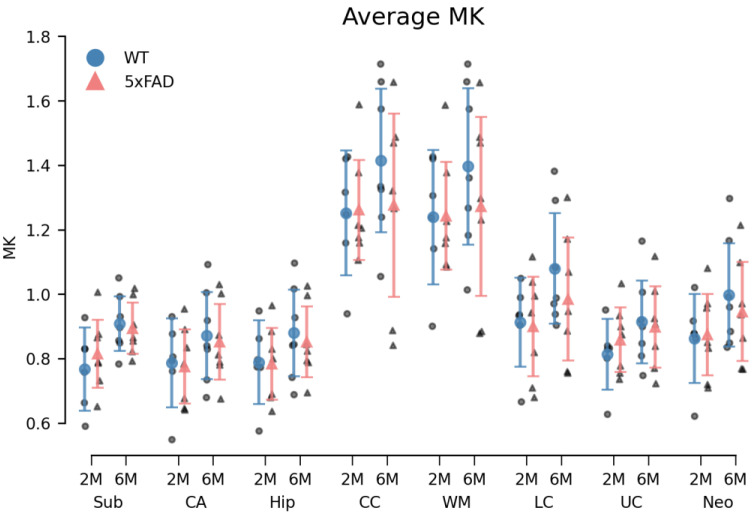
6M 5xFAD brains exhibited decreased averaged mean kurtosis (MK) in all regions of interest (ROIs). While the differences in MK between genotypes of 2-month-old (2M) brains varied between ROIs, a small but consistent decrease was observed in the 6-month-old (6M) 5xFAD brains compared to WT brains. This difference was not statistically significant. An age-related increase was observed in the ROIs of both genotypes, although less pronounced in 5xFAD brains. This age difference was not statistically different either. Group means with 95% confidence intervals are superimposed on the individual observations. Sub = subiculum, CA = cornu ammonis of the hippocampal formation, hip = hippocampus, CC = corpus callosum, WM = white matter, LC = lower cortex, UC = upper cortex, and neo = neocortex.

**Figure 8:**
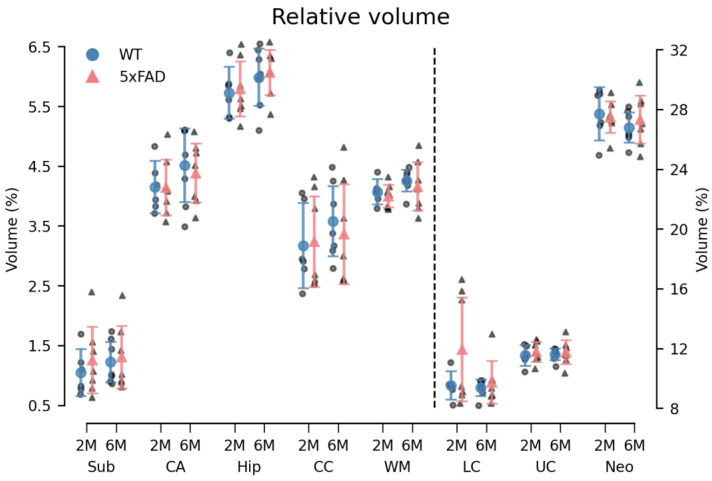
The relative volume of all ROIs was unchanged in 5xFAD brains. No difference was observed in the relative volume of the ROIs between genotypes. A statistically significant age × ROI interaction was observed. However, the simple main effects analysis found no statistically significant effect of age in any of the ROIs. The dotted line indicates that LC, UC, and neo are plotted against the right y-axis. Group means with 95% confidence intervals are superimposed on the individual observations. 2M = 2-month-old, 6M = 6-month-old. Sub = subiculum, CA = cornu ammonis of the hippocampal formation, hip = hippocampus, CC = corpus callosum, WM = white matter, LC = lower cortex, UC = upper cortex, and neo = neocortex.

Overall, a small, systematic decrease in MD of 5xFAD brains was observed in all ROIs in both age groups (Figure 6). For the 6M 5xFAD brains, the MK was similarly decreased compared to WT brains (Figure 7). However, none of these differences were statistically significant. An age-related increase was observed in both dMRI metrics (Figure 6 and Figure 7). No differences in the relative ROI volume were observed between the genotypes across age groups (Figure 8).

#### 3.3.1. Diffusion Kurtosis Imaging

The permutational three-way mixed ANOVA of the MD metric revealed a statistically significant difference between the ROIs (F(7161) = 75.51, *p* < 0.000). However, neither genotype (F(1,23) = 2.98, *p* = 0.100), age (F(1,23) = 1.67, *p* = 0.216), nor the genotype × age × ROI interaction (F(7161) = 0.25, *p* = 0.973) were statistically significant. Correspondingly, a statistically significant difference in MK was observed between the ROIs (F(7161) = 102.98, *p* < 0.000), while no statistically significant main effects of genotype (F(1,23) = 0.22, *p* = 0.647), age (F(1,23) = 3.06, *p* = 0.097) or genotype × age × ROI interaction (F(7161) = 0.36, *p* = 0.919) were observed (Figure 6 and Figure 7).

#### 3.3.2. Volumetrics

The relative volume of the ROIs was statistically different (F = (7161) = 1882.63, *p* < 0.000). However, as for the DKI parameters, neither the main effects of genotype (F(1,23) = 0.81, *p* = 0.384) and age (F(1,23) = 0.10, *p* = 0.759) nor the genotype × age × ROI interaction (F(7161) = 1.34, *p* = 0.226) were statistically significant (Figure 8). A statistically significant age × ROI interaction (F(7161) = 2.34, *p* = 0.025) was observed. However, a simple main effects analysis revealed no statistically significant differences between age groups in any of the ROIs. See Figure S4 for absolute ROI volumes.

## 4. Discussion

By combining immunohistochemistry, functional metabolic mapping, and diffusion MRI, we provide a comprehensive view of AD pathology in the 5xFAD model that highlights glial disturbances. Our multimodal analysis integrates functional and structural levels to better understand the complex interplay between neuropathological markers, metabolic shifts, and microstructural changes in AD. We show regional and age-dependent expression of AD markers, while the functional metabolic mapping reveals intricate details in cell-specific metabolic pathways, particularly demonstrating glial metabolic alterations. dMRI offers a means to assess brain microstructure and volumetrics, which seem to be unchanged in the 5xFAD brains compared to WT controls.

### 4.1. Age-Dependent Neuropathological and Microstructural Changes in 5xFAD Mice

Our study demonstrates age-dependent increases in Aβ, GFAP, and IBA1 levels in 5xFAD mice, with marked differences in specific brain regions. At 2 months, 5xFAD mice exhibited elevated Aβ levels in the subiculum compared to WT controls. By 6 months, Aβ accumulation extended to the subiculum, upper cortex, lower cortex, and white matter, aligning with previous reports that describe progressive Aβ deposition in the 5xFAD mouse model [8]. These findings suggest that early detection of Aβ in the subiculum could be a valuable marker for monitoring AD progression. Our data align with studies showing significant differences between the upper and lower cortex in 6M 5xFAD mice. Maharjan et al. found more Aβ plaques in the lower cortex layers at 4, 7.5, and 12 months [40]. In contrast to their study, our work includes early detection (2M) of Aβ, GFAP, and IBA1. Our findings reveal no differences in these markers between the upper and lower cortex in 2M 5xFAD mice, contrasting with the significant differences observed in 6M 5xFAD mice.

Similarly, GFAP levels were significantly increased in the subiculum and white matter of 2M 5xFAD mice, indicating early astrocytic activation, consistent with previous studies identifying GFAP as a potential early hallmark of AD [41,42]. At 6 months, GFAP elevation was observed across multiple regions, including the upper and lower cortex, further supporting the progressive nature of astrocytic involvement in AD. In contrast, IBA1 levels, indicative of microglial activation, did not differ between 2M 5xFAD and WT mice. However, by 6 months, significant increases were noted in the subiculum, cortex, and white matter. This temporal pattern suggests that reactive microglia may emerge later in the disease course, likely as a secondary response to accumulating Aβ, corroborating earlier findings [43].

Notably, MBP levels in the white matter of WT mice were lower at 6M compared to 2M. This finding is consistent with studies that report a relatively greater age-associated decline in white matter volume or the presence of white matter volume loss without a corresponding loss in grey matter ([44] and references therein). The observed decrease in MBP levels in the subiculum and lower cortex of 6M 5xFAD mice, but not at 2 months, points to age-dependent white matter degradation in AD. While early MBP reduction was reported in a different AD model [45], our findings imply that MBP may not serve as an early biomarker in 5xFAD mice, potentially due to model-specific differences or methodological variations in MBP quantification.

In summary, this study not only confirms the progressive accumulation of Aβ, GFAP, and IBA1 in the 5xFAD model but also highlights the differential vulnerability of specific brain regions to AD pathology. The use of triple fluorescence staining techniques provides a comprehensive view of the spatial and temporal dynamics of these markers, reinforcing the utility of the 5xFAD mouse model in studying AD-related processes.

The DAPI staining results provide intriguing insights into cellular dynamics in different brain regions of 5xFAD mice. Our findings show no visual differences in DAPI staining between 5xFAD and WT mice under fluorescence microscopy, suggesting similar nuclear density across genotypes. However, the quantitative analysis paints a more nuanced picture, highlighting significant regional and age-dependent differences in cell density that warrant further exploration. The lack of significant differences in DAPI cell counts in the subiculum between 5xFAD and WT mice across age groups contrasts with the well-documented neurodegenerative processes in AD, where substantial neuronal loss is often observed in regions such as the hippocampus formation [46]. The observed increases in DAPI cell counts in both the upper and lower cortex of 6M 5xFAD and WT mice compared to their 2M counterparts align with reports of gliosis—a reactive increase in glial cells—in response to accumulating Aβ plaques [43]. The higher DAPI cell count in 2M WT mice compared to 2M 5xFAD mice, particularly in the cortex, suggests early cortical cellular deficits in 5xFAD mice, potentially due to early neuroinflammatory processes. The significant increase in DAPI cell count from 2 to 6 months in both 5xFAD and WT mice across several regions, including the upper and lower cortex and white matter, is consistent with developmental changes and aging processes. In WT mice, this increase could reflect normal age-related gliogenesis or synaptic pruning, while in 5xFAD mice, it might indicate reactive gliosis in response to progressive Aβ accumulation [47]. However, the lack of significant difference in DAPI cell counts between 6M 5xFAD and WT mice in the upper cortex suggests that early deficits may have plateaued, or that compensatory mechanisms, such as increased glial proliferation, might be at play [48,49]. However, DAPI staining does not differentiate between cell types, and further investigation is needed to determine whether the increased cell count reflects an increase in neurons, glia, or other cell types.

### 4.2. Localized Energy Substrate-Specific Alterations in 5xFAD Mice

Here, we observed region-specific disruptions in brain energy metabolism in 6M 5xFAD mice. While the glycolytic activity and mitochondrial metabolism of glucose ([U-^13^C]glucose) appeared preserved in the cerebral cortex, significant hypometabolism of glucose was evident in the hippocampus, as indicated by reduced ^13^C-labeling in TCA cycle intermediates such as citrate, succinate, and malate. This selective glucose hypometabolism in the hippocampus aligns with the findings of Andersen et al. [9], who reported early disruptions in synaptic and astrocyte metabolism in the hippocampus of 5xFAD mice, and highlights the hippocampus as a particularly vulnerable region in early AD pathology, which is further supported by our immunohistochemical observations. Conversely, when [1,2-^13^C]acetate, a substrate preferentially metabolized by astrocytes [36], was used, we found impaired TCA cycle activity in the cortical region of 5xFAD mice. The reduced ^13^C-enrichment in succinate, fumarate, and malate in the cortical slices, but not in the hippocampal slices, suggests a region-specific dysregulation of astrocyte metabolism. These findings align with growing evidence that astrocytes exhibit metabolic alterations in AD, which may exacerbate neuronal energy deficits and contribute to disease progression.

Our results echo the findings of previous reports [9,12,13,47,50] that demonstrated that metabolic dysregulation, particularly in astrocytes, plays a critical role in AD. They suggested that astrocytic energy metabolism is crucial for maintaining neuronal health, and its disruption could lead to the progression of neurodegeneration. Additionally, Salcedo et al. [13] highlighted that altered astrocytic metabolism, especially in the context of impaired glutamate uptake and processing, could lead to excitotoxicity, further contributing to neuronal damage in AD.

The observed preservation of glucose metabolism in the cortex, despite marked astrocytic metabolic deficits, reflects the complex cellular interplay in AD-affected regions.

The reduced glucose metabolism in the hippocampus, alongside preserved glycolysis in the cortex, suggests region-specific vulnerabilities that may underpin the differential progression of AD pathology. This notion is further supported by Andersen et al. [9], who demonstrated that hippocampal disruptions in metabolism precede synaptic degeneration, indicating that metabolic disturbances could be early events driving AD progression in this region.

### 4.3. Altered Glutaminolysis Dynamics in 5xFAD Cortex

We also investigated glutamine metabolism by incubating cortical and hippocampal slices with [U-^13^C]glutamine. Interestingly, while glutamine uptake appeared unchanged in both the cortical and hippocampal slices of 5xFAD mice, we observed increased ^13^C-enrichment in metabolites derived from glutaminolysis in the cortical slices of 5xFAD mice. This finding suggests an upregulation of glutaminolysis in the cortex, potentially reflecting an adaptive response to increased metabolic demands by activated microglia, which are known to rely on glutaminolysis to meet their energy requirements under inflammatory conditions [39]. This metabolic reprogramming of microglia has been implicated in AD pathology and is consistent with the notion that microglial activation plays a significant role in altering the local metabolic environment to support its heightened activity.

Although glutamine is largely taken up and metabolized by neurons to support glutamate and GABA synthesis as well as energy metabolism [38], the increased glutaminolysis observed in the cortex contrasts with the preserved metabolism of glucose (the necessary neuronal energy substrate), suggesting a shift in the metabolic strategy of cortical cells, particularly microglia and astrocytes. This shift may serve to meet the increased energy demands associated with neuroinflammation and neuronal stress. These findings are in line with the study by Andersen et al. (2021) [12], which reported elevated cortical glutamine metabolism at a late stage of disease progression (8M) in the 5xFAD model. This shift in metabolic strategy in the cortex, characterized by enhanced glutaminolysis, is consistent with recent reports of altered microglia metabolism in neurodegenerative disorders, including upregulated glutaminolysis, to support their pro-inflammatory and neurotoxic roles [51]. The selective upregulation of glutaminolysis in the 5xFAD cortex, despite maintained glucose metabolism, suggests a complex interplay between different cell types in response to AD pathology, where microglial activation may play a significant role in altering the local metabolic environment to support its heightened activity.

These findings have several important implications. First, the region-specific metabolic disruptions observed in 5xFAD mice underscore the heterogeneity of metabolic alterations in AD, with the hippocampus being particularly susceptible to glucose hypometabolism, a major hallmark of AD pathology. The preserved glycolysis in the cortex, alongside impaired astrocytic metabolism, suggests that while neurons may maintain their metabolic function to some extent, astrocyte dysfunction could still contribute to overall cortical pathology. This is particularly relevant in the context of recent literature, which has emphasized the critical role of astrocytes in maintaining neuronal health and the consequences of their dysfunction in neurodegenerative diseases like AD.

The increased glutaminolysis observed in the cortex suggests that microglial activation and the associated metabolic reprogramming could be early events in AD, potentially preceding or coinciding with neuronal dysfunction. This aligns with previous studies [13,50,51,52] that reported that metabolic reprogramming in glial cells, particularly microglia and astrocytes, plays a critical role in the early stages of AD and may contribute to the progression of the disease.

These results highlight the importance of examining cell-specific and region-specific metabolic pathways in understanding AD pathology. The differential metabolic profiles between the cortex and hippocampus in 5xFAD mice point to the need for targeted therapeutic strategies that address these distinct metabolic disturbances. Future research should aim to elucidate the precise mechanisms underlying these metabolic shifts and explore how they contribute to the progression of AD, potentially opening new avenues for therapeutic intervention.

### 4.4. Impact of Age and Genotype on dMRI and Volumetric Measures in the 5xFAD Model

Diffusion MRI (dMRI) is widely regarded as a promising radiological method for the early detection of neurodegenerative diseases and for monitoring disease progression and treatment efficacy [53]. While dMRI methods suitable for human brain imaging exist [54], animal models of AD will continue to be valuable for the investigation of disease mechanisms and experimental treatments. The AD mouse model used here has previously been investigated using dMRI [40,55].

Similar to our study, in [40], no changes were detected by dMRI in most brain regions except for the cortex, where FA was found to be reduced in 5xFAD mice compared to WT. In the landmark paper by Johnson et al. [55], dMRI revealed reduced connectivity in brains from 5xFAD mice (5xFADBXD77). However, this finding was based on data from 150 specimens imaged at a higher spatial resolution (25 µm) than what was achievable on our system.

Our inability to detect differences between groups is therefore not entirely surprising. It is, however, of concern that even when using the sensitive DKI technique on ex vivo samples, detection capability is limited. Nevertheless, the lack of findings in our MRI investigation may not only be due to limited sensitivity. It should be noted that the samples in the present study were prepared somewhat differently than samples typically used for ex vivo MRI studies. Therefore, we cannot rule out that the use of sucrose immersion had reduced tissue water mobility to such an extent that the DKI method lost the microstructural sensitivity for which it is known. This was done here to ensure that dMRI was performed on the same samples as the histological investigations. While our dMRI findings do not reach significance, the fact that immunohistochemical changes were observed does lend support to the genotype-dependent tendencies seen in the dMRI data.

Cohort studies show dMRI to be capable of differentiating between controls and AD patients [54,56,57]. This is certainly of value for studying typical disease progression or treatment/intervention effects in larger groups. However, our study indicates that even under ideal conditions (ex vivo, high field MRI), dMRI methods may lack the sensitivity to detect alterations in smaller groups. Consequently, dMRI methods still lack the sensitivity needed for early disease detection in the individual patient. As it stands, our study shows that metabolomic changes precede MRI-visible microstructural alterations, but that such metabolic changes are detectable using other methods. This is important for the development and refinement of biomarkers in early AD.

## 5. Conclusions

The data presented demonstrate the intricate relationship between metabolic dysregulation, microglial activation, and microstructural abnormalities in the progression of AD. Our findings of region-specific metabolic alterations and enhanced glutaminolysis in the 5xFAD cortex suggest potential metabolic targets for therapeutic intervention. Future studies should aim to elucidate the mechanistic underpinnings of these metabolic shifts and explore their implications for AD progression and treatment. Integrating advanced imaging techniques with metabolic and molecular analyses will be crucial in advancing our understanding of AD pathology and developing effective therapeutic strategies.

## Data Availability

Data can be provided upon reasonable request by contacting the authors.

## Supplementary Materials

The following supporting information can be downloaded at: https://www.mdpi.com/article/10.3390/biom14101294/s1: Table S1: Overview of the primary and secondary antibodies used in the study; Figure S1: MRI segmentation of the regions of interest; Figure S2: Quantification of MBP in the brains of 5xFAD and WT mice of two ages; Figure S3: Hippocampal glucose metabolism is selectively lower in 6-month-old 5xFAD brains; Figure S4: The absolute volume of the regions of interest (ROIs).
