# Supplementary material for: Comprehensive Analysis of the 5xFAD Mouse Model of Alzheimer’s Disease Using dMRI, Immunohistochemistry, and Neuronal and Glial Functional Metabolic Mapping
Source: Biomolecules. 2024 Oct 13;14(10):1294. doi: 10.3390/biom14101294 (PMC11505609; doi:10.3390/biom14101294)
Supplement: Supplementary file 1 [file biomolecules-14-01294-s001.zip › biomolecules-3218049-supplementary.pdf]

Article

Comprehensive Analysis of the 5xFAD Mouse Model of Alzheimer's Disease Using dMRI, Immunohistochemistry, and Neuronal and Glial Functional Metabolic Mapping

Emil W. Westi[1#], Saba Molhemi[2#], Caroline Termøhlen Hansen[1], Christian Stald Skoven[2], Rasmus West Knopper[2,3], Dashne Amein Ahmad[1], Maja B. Rindshøj[1], Aishat O. Ameen[1], Brian Hansen[2], Kristi A. Kohlmeier[1], and Blanca I. Aldana [1,*]

Supplementary Materials:

Table S1: Overview of the primary and secondary antibodies used in the study.

Figure S1: MRI segmentation of the regions of interest;

Figure S2. Quantification of DAPI in the brain of 5xFAD and WT mice of two ages;

Figure S3. Hippocampal glucose metabolism is selectively lower in 6M 5xFAD brains;

Figure S4. The absolute volume of the regions of interest (ROIs).

Methods and materials:

Table S1: Overview of the primary and secondary antibodies used in the study.

Antigen/Specificity	Host	Fluorescence	Working dilution	Catalog no.	Source
Primary antibody					
Aβ	Mouse	-	1:1000	MABN10	Sigma-Aldrich
GFAP	Chicken	-	1:1000	Ab4674	Abcam
IBA1	Goat	-	1:1000	Ab5076	Abcam
MBP	Rabbit	-	1:1000	Ab218011	Abcam
Secondary antibody					
Anti-mouse	Donkey	Alexa Fluor 594	1:1000	A21203	Fischer Scientific
Anti-mouse	Goat	Alexa Fluor 488	1:1000	A21202	Fischer Scientific
Anti-chicken	Goat	Alexa Fluor 594	1:1000	A11042	Fischer Scientific
Anti-rabbit	Goat	Alexa Fluor 488	1:1000	A11008	Fischer Scientific
Anti-goat	Donkey	Alexa Fluor 488	1:1000	A1105	Fischer Scientific
Fluorescent counterstain					
DAPI		Em 461	1:30.000	D9542	Sigma-Aldrich

Source=commercial source; fluorescence= fluorescence label/fluorophores

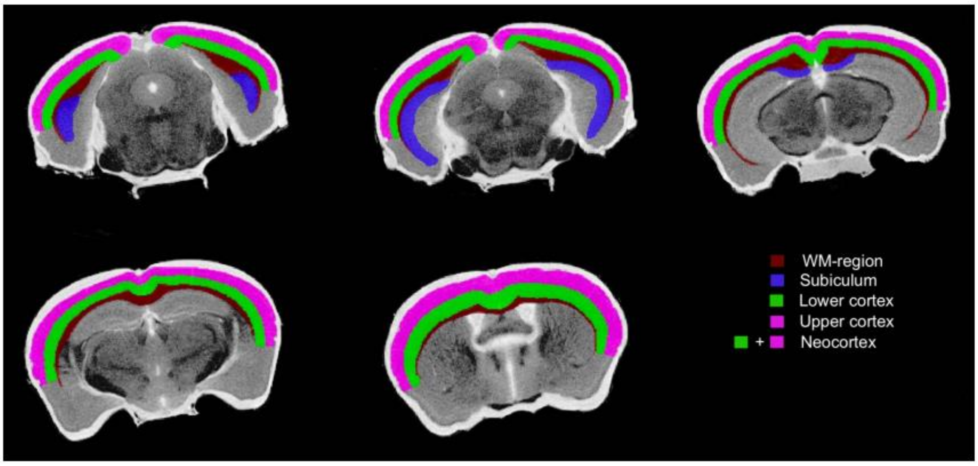

Figure S1: MRI segmentation of the regions of interest. *All regions of interest (ROI) were manually outlined in 2D coronal cross-sections from the T2-weighted images, using ITK-SNAP aided by standard mouse brain atlases. The ROIs were: neocortex, upper and lower cortical layer, subiculum and a general WM-region. To avoid partial volume effects this WM-region consisted of corpus callosum, cingulum, external capsule and the dorsal hippocampal commissure.*

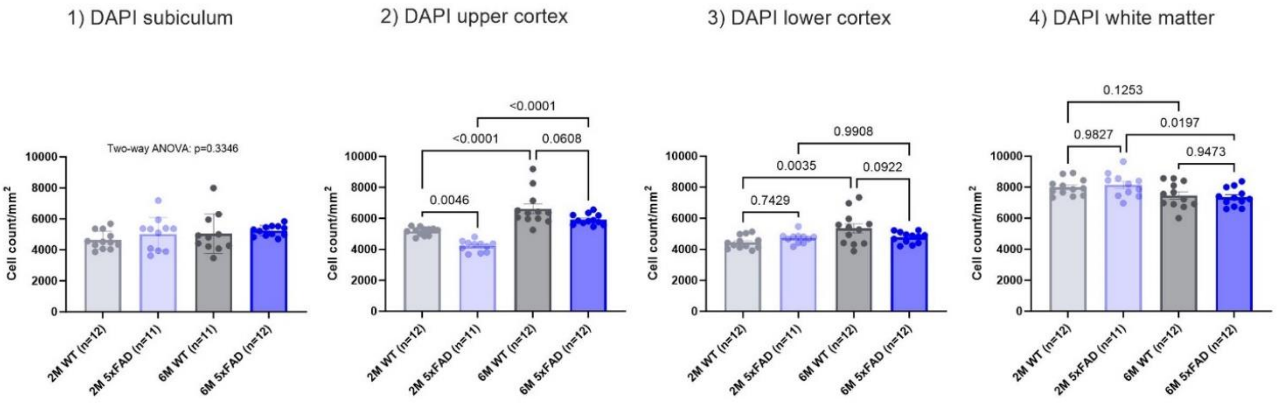

***Figure S2. Quantification of DAPI in the brain of 5xFAD and WT mice of two ages*.** The four bar graphs cover four different brain regions: 1) subiculum, 2) upper cortex, 3) lower cortex, and 4) white matter. Each bar graph represents populations of DAPI cell count in 2M 5xFAD, 2M WT, 6M 5xFAD and 6M WT mice. The bar graphs are presented as cell count/mm2, measured in images of brain slices with the fluorescence staining of DAPI. The number of brain slices is indicated by the letter n. The light grey bar indicates brain slices of 2M WT mice. The light blue bar indicates brain slices of 2M 5xFAD mice. The dark grey bar indicates brain slices of 6M WT mice. The dark blue bar indicates brain slices of 6M 5xFAD mice. The level of significance is presented above the bars with asterisk. 1) In subiculum, statistical analysis showed no significant difference between the groups (ANOVA: p=0.3346). 2) In upper cortex, a significant difference was seen in the statistical analysis (ANOVA: p<0.0001) between 2M 5xFAD and 2M WT (Tukey's test: p=0.0046), between 2M 5xFAD and 6M 5xFAD (Tukey's test: p<0.0001), and between 2M WT and 6M WT (Tukey's test: p<0.0001). No significant difference was seen between 6M 5xFAD and 6M WT (Tukey's test: p=0.0608). 3) In the lower cortex, the ANOVA analysis showed significant difference (ANOVA: p=0.0055) between 2M WT and 6M WT (Tukey's test: 0.0035). No significant difference was seen between 2M 5xFAD and 2M WT (Tukey's test: p=0.7429), between 6M 5xFAD and 6M WT (Tukey's test: p=0.0922), and between 2M 5xFAD and 6M 5xFAD (Tukey's test: p=0.9908). 4) In white matter, a significant difference was observed (ANOVA: p=0.0061) between 2M 5xFAD and 6M 5xFAD (Tukey's test: p=0.0197). No significant difference was seen between 2M 5xFAD and 2M WT (Tukey's test: p=0.9827), between 6M 5xFAD and 6M WT (Tukey's test: p=0.9473), and between 2M WT and 6M WT (Tukey's test: p=0.1253).

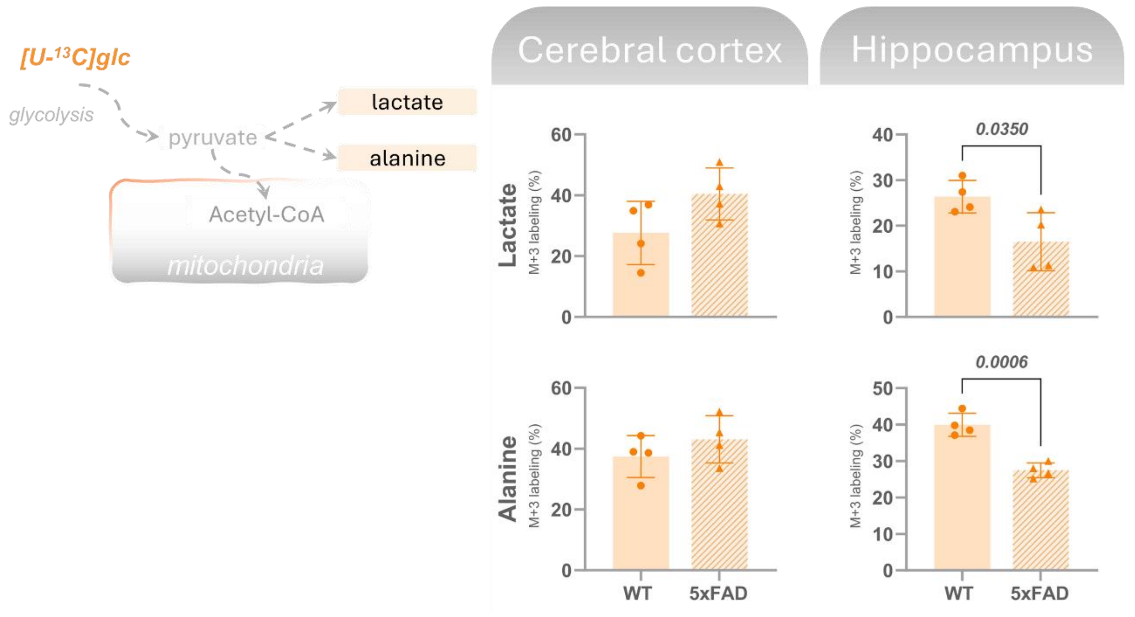

Figure S3. Hippocampal glucose metabolism is selectively lower in 6M 5xFAD brains. *A) Glycolytic metabolism of [U-^{13}C]glucose (glc) gives rise to ^{13}C-enrichment (detected by GC–MS) in lactate and alanine (M+3) from labeled pyruvate in acutely isolated slices from B) cerebral cortex or C) hippocampus incubated with the labeled substrates for 60 min. In the cerebral cortex, overall maintained ^{13}C-incorporation in products derived from [U-^{13}C]glucose metabolism was observed while lower ^{13}C enrichment in both lactate and alanine was found in the hippocampus of 5xFAD mice compared to wild-type (WT) controls. Values represent mean (±) SD (n = 4 animals). The statistical significance (determined with Student's unpaired t-test) is presented with p values above the bars with significant values in bold.*

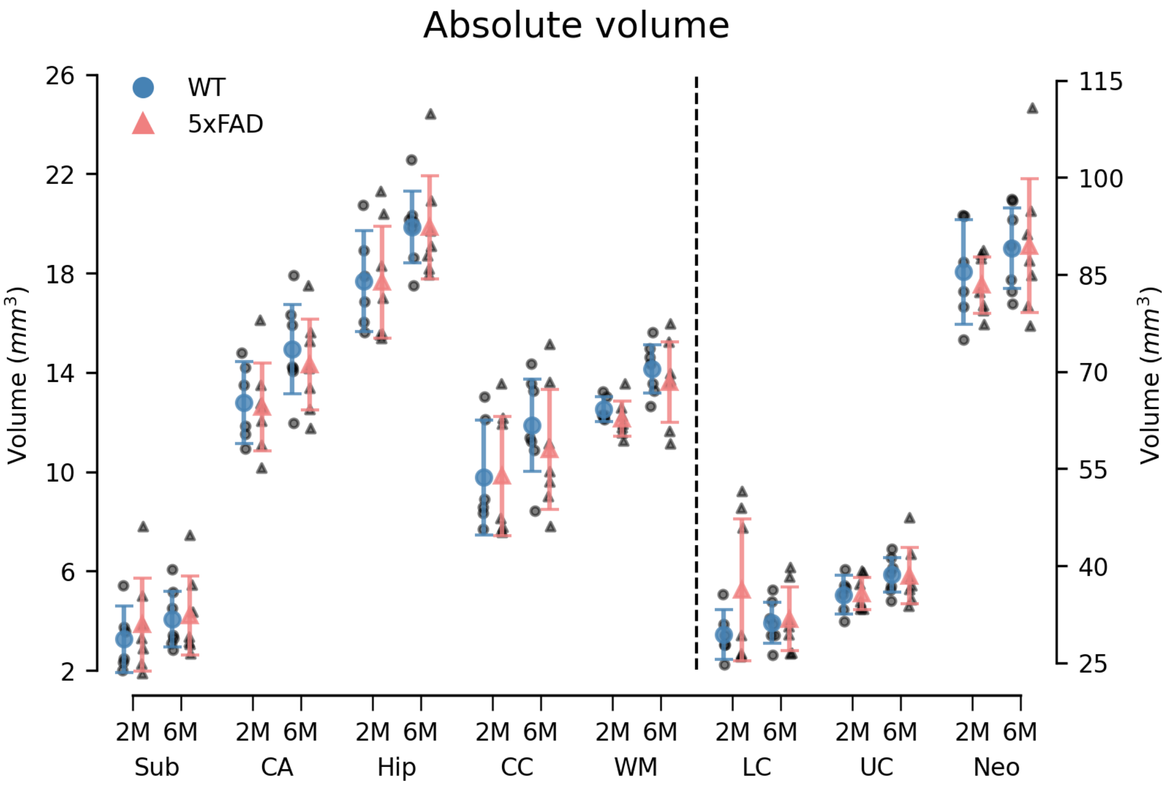

Figure S4. The absolute volume of the regions of interest (ROIs). *The absolute ROI volume estimates are normalized to the whole-brain volumes and presented as the relative ROI volume in Figure 8.*
